# Association of Helicobacter pylori antibodies and severity of migraine attack

**Published:** 2015-07-06

**Authors:** Behnaz Ansari, Keivan Basiri, Rokhsareh Meamar, Ahmad Chitsaz, Shahrzad Nematollahi

**Affiliations:** 1 Department of Neurology, School of Medicine, Isfahan University of Medical Sciences, Isfahan, Iran; 2 Isfahan Neurosciences Research Center, Al-zahra Hospital, Isfahan University of Medical Sciences AND Department of Medical Sciences, School of Medicine, Islamic Azad University, Najafabad Branch, Isfahan, Iran; 3 Department of Biostatistics and Epidemiology, School of Public Health, Isfahan University of Medical Sciences, Isfahan, Iran

**Keywords:** Helicobacter Pylori, Migraine, Head Pain

## Abstract

**Background: **Recent studies have shown a positive correlation between Helicobacter pylori infection and migraine headache. The aim of this study was to evaluate the role of H. pylori infection in migraine headache with (MA) and without aura (MO).

**Methods:** This is a case-control study containing information on 84 patients (including MA, MO) and 49 healthy individuals**. **The enzyme-linked immunosorbent assay (ELISA) test was used to measure immunoglobulin G (IgG,) immunoglobulin M (IgM) titer in two groups. Headache severity was evaluated according to Headache Impact Test (HIT6) questionnaire.

**Results: **Mean ± SD of IgM antibody in Migrainous patients 26.3 (23.1) showed significantly difference with control group 17.5 (11.2) (P = 0.004). In addition, the mean ± SD HIT6 in Migrainous patients differed significantly between MA and MO groups 65.5 (4.7), 54.9 (5.3) respectively, P < 0.001)**.** The only significant correlation was found for IgG antibody and HIT6 in MA patients (r = 0.407, P = 0.011) and MO group (r = 0.499, P = 0.002). The risk of migraine occurrence in patients did not significantly associate with the level of IgG and IgM antibodies.

**Conclusion: **The results give a hope that definite treatment and eradication of this bacterium could be a cure or to reduce the severity and course of migraine headaches.

## Introduction

Migraine is a common primary headache disorder with the prevalence of nearly 15% in Western societies.^1^

Migraine is divided into two main categories: migraine with aura (MA), which patients experience transient visual or sensory symptoms (including flickering lights, spots, or pins that develop 5-20 min before attacks), and migraine without aura (MO).^2^^,^^3^

Many factors such as genetics, food and nutrients, sleep disorders, environmental factors such as noise, light, and humidity, menstruation, severe trauma, and alcohol even total fat-free mass have been reported as precipitating factors and the possible causes of migraine headaches.^4^^,^^5^

In the recent years, the role of infections and also the impact of digestive system disorders on migraine have gained more attention.

Migraine headaches are reported frequently by patients with various gastrointestinal symptoms.^6^^-^^8^ However, in last few years, researches have focused on the role of Helicobacter pylori activity in the pathogenesis of migraine.

According previous reports, relationship between H. pylori and both MA, MO has been reported.^9^^-^^12^ It is postulated that recurrent headache secondary to H. pylori infection could be the result of systemic vasospastic effects of pro-inflammatory substances which released by infected gastric mucosa.^10^^,^^13^

It has been also shown that eradication of H. pylori significantly reduces the frequency, intensity, and duration of migraine attacks.^9^^,^^14^^-^^17^

Since reducing the severity and course of migraine headaches by definite treatment and consequently eradication of the H. pylori infection shows promising results,^12^ the current study is designed to evaluate the role of H. pylori infection both in MO or MA patients.

## Materials and Methods

The present case-control study contains information of 84 patients of MA and MO that were diagnosed by experienced neurologist, according to the International Headache Society criteria [Headache Classification Committee of the International Headache Society (IHS)]^18^ referring to an educational hospital in Isfahan, Iran (Al-Zahra).

The inclusion criteria for the patients were age between 15 and 50 years, without gastrointestinal symptoms (such as pyrosis, epigastric pain, belching, bloating) or receiving any nonstandard medication for H. pylori, and physical and mental ability to give written consent form.

Controls were 49 randomly selected companions of non-migrainious patients referring to the Al-Zahra hospital at the about same time as cases.

In the control group, after matching for sex and age with patients group, were included the person should not have any history of migraine headaches. Group matching was done according to educational level, marital status, geographical origin, and socio-economic status. In order to find the appropriate sample size, we used the H. pylori prevalence among cases to be 40%.^10^

The data on age, sex, antibodies including immunoglobulin G (IgG,) immunoglobulin M (IgM) titer (by Enzyme Linked Immunosorbent Assay or ELISA) gathered in all participants in two groups. Furthermore, headache severity was evaluated according to Headache Impact Test (HIT6) questionnaire.^19^

Statistical software SPSS for Windows (version 18.0, SPSS Inc., Chicago, IL, USA) was used for all statistical calculations. The comparison of clinical characteristics of study groups with regarding measured variables was achieved by t-tests. Associations between H. pylori antibodies and severity of headache were estimated using Pearson correlation coefficient. P ≤ 0.050 was considered in all tests as a significant level.

## Results

Table 1 represents the main characteristics of the study groups. Totally, there were included 84 migraine patients in the case group and 49 healthy individuals in the control group. The mean ± SD age is 35.8 ± 11.1 and 33.4 ± 18.9 for case and control group, respectively. Mean ± SD of IgM antibody in Migrainous patients 26.3 (23.1) showed significantly difference with the control group 17.5 ± 11.2 (P = 0.004) but such result did not observe in IgG titer antibody. In addition, the mean ± SD HIT6 in Migrainous patients differed significantly between MA and MO groups 65.5 ± 4.7, 54.9 ±5.3, respectively, (P < 0.001).

In order to find the possible correlations between MA and MO group with regard to different variables, the Pearson correlation coefficient was utilized. The only significant correlation was found for IgG antibody and HIT6 in MA patients (r = 0.407, P = 0.011) and MO group (r = 0.499, P = 0.002).

In the next step based on the laboratory test results (17), H. pylori antibodies divided to “Normal” category (≥ 30 UR/ml for IgG, and ≥ 40 ml/g for IgM), and “High” category (< 30 UR/ml for IgG, and < 40 ml/g for IgM) in migrainous patients.

Table 2 represents the relationship between the aforementioned categories with the severity of headache in the patients group. The results of this table show that a statistically significant difference exist between normal level and high level of IgG antibody with regard to the severity of headache (P = 0.002).

Table 3 shows the results of a logistic regression model with the occurrence of MA attacks as the dependent variable response. Based on this table, the risk of migraine occurrence in patients did not significantly associate with the level of IgG and IgM antibodies.

**Table 1:** Baseline characteristic of migraine patients and healthy individuals according to Helicobacter pylori antibody and Headache Impact Test (HIT6) questionnaire

**Variables (mean ± SD)**	**Healthy control ** **(n = 49)**	**Migraine patients**			**P**	
		**MA (n = 43)**	**MO (n = 36)**	**Total (n = 79)**	**Case versus ** **control**	**MO versus ** **MA**
Age	33.4 ± 18.9	33.5 ± 11.3	37.6 ± 10.4	35.8 ± 11.1	0.375	0.093
HIT6	-	65.9 ± 4.7	59.4 ± 5.3	62.3 ± 6.0	-	< 0.001
IgG (UR/ml)	34.8 ± 40.4	33.1 ± 35.4	29.0 ± 34.2	30.9 ± 34.2	0.570	0.593
IgM (UR/ml)	17.5 ± 11.2	28.1 ± 24.6	25.2 ± 23.3	26.3 ± 23.1	0.004	0.585

HIT: Headache Impact Test Questionnaire; IgG: Immunoglobulin G; IgM: Immunoglobulin M; MO: Migraine without aura; MA: Migraine with aura; SD: Standard deviation

**Table 2:** The relationship between headache severity and antibody levels in migrainous patients

**Antibodies level**	**HIT6 (mean ± SD)**	**P**
IgG (UR/ml)		
Normal level	61.1 **±** 5.5	0.002
High level	65.7 **±** 6.0	
IgM (UR/ml)		
Normal level	62.1 **±** 6.0	0.364
High level	63.6 **±** 5.6	

HIT: Headache impact test questionnaire; IgG: Immunoglobulin G; IgM: Immunoglobulin M; SD: Standard deviation

**Table 3:** Correlation between antibodies level with occurrence of migraine attacks using logistic regression

**Variables**	**P**	**OR**	**B**	**95% CI for OR**	
				**Lower**	**Upper**
IgG	0.160	0.37	-0.972	0.098	1.468
IgM	0.458	1.67	0.517	0.428	6.570

OR: Odds ratio; CI: Confidence interval; IgG: Immunoglobulin G; IgM: Immunoglobulin M

## Discussion

Based on the literature review, this is the first study attempting to find a correlation between the severity of headache (in terms of HIT6) and H. pylori antibody levels in migraneous patients either with or without aura.

Our results revealed a strong correlation between IgG antibody and the severity of headache between both migraine subgroups. However, no statistically significant difference has been observed in levels of IgG in MA vs. MO groups, as well as in patients versus controls. This finding has been supported by some researches,^20^^,^^21^ however, some authors argued that compared to the general population, higher IgG antibody titer is seen in migraineous patients.^10^^,^^12^^,^^14^ One reason for seeing such controversial result is that we used matching based factors that may have an effect on H. pylori infection including socio-economic status.^22^ Moreover, literature used a variety of control types that differed with our controls in many ways.

However, the significance difference was found (P = 0.004) in IgM antibody titer against H. pylori in our migrainous patients compared to control groups. This finding has shed light to the importance of studying active infection with this bacterium in the etiology of migraine headaches. Previous studies concluded that active H. pylori infection is strongly related to the occurrence and severity of migraine headaches, and H. pylori treatment reduces severity and frequency of the migraine attacks significantly.^19^^,^^23^ Gasbarrini et al. showed that treatment on patients in whom with the active form of H. pylori, a significant difference was observed in reducing frequency, intensity, and duration of migraine attacks.^24^ As a result, the active H. pylori infection is strongly related to the outbreak and severity of migraine headaches, and proper treatment against H. pylori could diminish obviously migraine headaches.

Hosseinzadeh et al. in a case-control study showed that the higher frequency of migraine headaches is observed in patients with gastrointestinal symptoms.^12^ Moreover, Gervil et al. performed two studies on 688 patients with gastrointestinal disorders in Italy found that a significant correlation between migraine and digestive disorders exists.^25^ This finding was further confirmed by other studies.^26^^,^^27^

The pathophysiological mechanism of chronic migraine has not been discovered yet. It is hypothesized that there is a possible involvement of more than one level of the nervous system. The central hypersensitivity of the trigeminal vascular complex increments excitability or decreases pain inhibitory mechanisms.^28^^,^^29^

It has been suggested that the pathogenic role of the H. pylori infection in migraine, based on a relationship between the host immune response against the bacterium and the chronic release of vasoactive substances. Postulated factors of the relationship between migraine and H. pylori infection included inflammation, oxidative stress, nitric oxide imbalance, or virulence of CagA-positive H. pylori strains.^10^^,^^17^^,^^30^

During the infection, the bacterium releases in the infected tissue toxins promoting the special cascade of events related to the host immune response alterations of vascular permeability.^31^^-^^33^

Other products included superoxide radicals and nitric oxide.^34^ Consequently, the resulting oxidative damage may be assessed as an aggregation of lipid peroxidation by products in the blood stream. Therefore, the prolonged oxidative injury caused by the persistent infection and the release of vasoactive substances might be involved in local cerebral blood circulation changes during migraine attacks.^35^ It has been also demonstrated that migraineous patients suffer from elevated plasma Ig levels. However, Ciancarelli et al. showed that H. pylori infection does not potentiate the plasma oxidative status and the systemic nitric oxide bioavailability of migraineuos patients. Therefore, they concluded that any specific correlation between H. pylori infection and migraine does not exist.^35^ In addition, in a case-control study was showed that lower nitrate levels have been found in migraneous patients without aura compared to controls. However, they concluded that the results do not support the role of oxidative stress in patients suffering from H. pylori infection and migraine.^36^

As a result, the infection of bacteria coincides with the severity and progression of the migraine headache; thus, the H. pylori infection can be regarded as one etiology of the migraine headaches.^12^

One of the major limitations of our study was the inability to provide the general inference based on these findings. The reason for this inability comes from the fact that the source population of the cases and controls could not be identified. Hence, drawing any rigid conclusions about these findings should be discouraged.

## Conclusion

According to the results of this study and similar researches, the existence of a correlation between IgG against H. pylori and severity changes in migraineous patients has been presented. Since IgG appears in the chronic pattern, association with the severity of migraine attack seems completely logical; but for better conclusion, further investigation should be designed. Furthermore, these results give a hope that definite treatment and eradication of this bacterium could be a cure or to reduce the severity and course of migraine headaches.^37^.

## Notes:

**How to cite this article: **Ansari B, Basiri K, Meamar R, Chitsaz A, Nematollahi Sh. Association of Helicobacter pylori antibodies and severity of migraine attack. Iran J Neurol 2015; 14(3): 125-9.
